# Transient Unexplained Severe Acute Hyperbilirubinaemia and Cholestasis in a Patient With Hereditary Spherocytosis

**DOI:** 10.1155/crhe/9398845

**Published:** 2026-02-13

**Authors:** Jennifer Richardson, Michael Johnston, Alison Sefcick, Karin Oien

**Affiliations:** ^1^ IMT Trainee West of Scotland, Queen Elizabeth University Hospital Glasgow, NHS Greater Glasgow and Clyde, Glasgow, UK, nhsggc.org.uk; ^2^ Consultant in Hepatology and Gastroenterology, Queen Elizabeth University Hospital Glasgow, NHS Greater Glasgow and Clyde, Glasgow, UK, nhsggc.org.uk; ^3^ Consultant Haematologist, Royal Alexandra Hospital, Paisley, NHS Greater Glasgow and Clyde, Glasgow, UK, nhsggc.org.uk; ^4^ Consultant Pathologist, Queen Elizabeth University Hospital Glasgow, NHS Greater Glasgow and Clyde, Glasgow, UK, nhsggc.org.uk

**Keywords:** acute cholestasis, haematology, hepatology, hereditary spherocytosis

## Abstract

Hereditary spherocytosis is an inherited red cell membrane disorder resulting in haemolytic anaemia. Recognised clinical manifestations include anaemia, jaundice, splenomegaly and gallstones. Here we describe the case of a 40‐year‐old male with hereditary spherocytosis presenting with severe hyperbilirubinaemia. Liver biopsy demonstrated features consistent with acute severe cholestasis. Despite extensive investigations for gallstone disease and other causes of liver pathology, no aetiology was identified. There are very few reports in the literature describing cases of profound unexplained jaundice in hereditary spherocytosis. Hereditary spherocytosis may be associated with idiopathic acute cholestasis. We report that the case was managed conservatively and spontaneously resolved.

## 1. Background

Hereditary spherocytosis is an inherited red blood cell membrane disorder which results in haemolytic anaemia. Due to deficiencies in structural proteins within the cell membrane, erythrocytes are unable to maintain their classical biconcave shape. This shape is essential for allowing a degree of flexibility within the red cell and the durability to withstand the mechanical and osmotic stresses of circulation. These abnormal red cells become spherical; spherocytes are removed from circulation by the spleen, resulting in extravascular haemolysis.

Clinical manifestations of this condition vary depending on the level of haemolysis. Key features are anaemia, splenomegaly and jaundice. Cholelithiasis is a common chronic complication; gallstones have been reported in 21%–63% of patients with hereditary spherocytosis [1]. The British Society for Haematology (BSH) cites symptomatic cholelithiasis as an indication for splenectomy in their 2011 guidelines for hereditary spherocytosis management. In some of these individuals, concurrent cholecystectomy is also recommended [2]. Hereditary spherocytosis is a heterogeneous disorder, and therefore it is important to identify the right patient cohorts who would benefit from splenectomy or cholecystectomy.

Jaundice may be present in individuals with hereditary spherocytosis even in the absence of anaemia, due to compensated haemolysis [3]. However, hyperbilirubinaemia accompanied by abnormal liver enzyme levels or bilirubin levels that are disproportionate to the apparent degree of haemolysis should prompt further evaluation. We detail our consideration of the numerous causes of elevated bilirubin in the sections below.

## 2. Case Presentation: History and Examination

Here we present the case of a 40‐year‐old male with acute cholestasis and severe hyperbilirubinaemia in the context of hereditary spherocytosis.

This patient first presented acutely to the general surgery department with a 5‐day history of right upper quadrant pain and nausea. He subsequently developed pale stools and dark‐coloured urine.

He had a background of hereditary spherocytosis diagnosed in his early twenties as part of a family screening process. He also had a history of anxiety and gout. A previous abdominal ultrasound had reported the presence of gallstones and splenomegaly. He had been referred to the general surgeons for consideration of splenectomy due to fatigue related to compensated haemolysis but had decided against surgery at that time.

A careful drug history revealed no culprit medication. There was no report of recent travel, recreational drug use or over‐the‐counter medications. He did not report alcohol excess.

Clinical examination revealed jaundice with mild epigastric and right upper quadrant tenderness. Initial blood tests showed bilirubin 341  μmol/L, alkaline phosphatase (ALP) 178U/L, alanine aminotransferase (ALT) 347 U/L, aspartate aminotransferase 361U/L, white blood cell count 13x10^9^/L and haemoglobin 140 g/L. Coagulation screen and renal function were unremarkable.

Over several days, the patient’s bilirubin continued to increment; he complained of anorexia, fatigue, nausea and generalised abdominal discomfort. He developed profound hyperbilirubinaemia with cholestatic liver enzymes. Bilirubin peaked at 1026  μmol/L (conjugated bilirubin 606  μmol/L, unconjugated 420  μmol/L), ALP 327 U/L, ALT 135 U/L and AST 69 U/L.

## 3. Case Presentation: Investigations and Differential Diagnosis

The degree of hyperbilirubinaemia was disproportionate to the apparent level of haemolysis seen in this patient with hereditary spherocytosis. A haemolysis screen demonstrated that haptoglobin was low at < 0.08 g/L, lactate dehydrogenase (LDH) was elevated at LDH 265 U/L, and reticulocytes were elevated at 427x10^9^/L. Although this was consistent with haemolysis, the haemoglobin remained within the reference range. In discussion with haematology, it was agreed that haemolysis alone would certainly not explain this acute rise in bilirubin and alternative causes should be sought.

**FIGURE 1:**
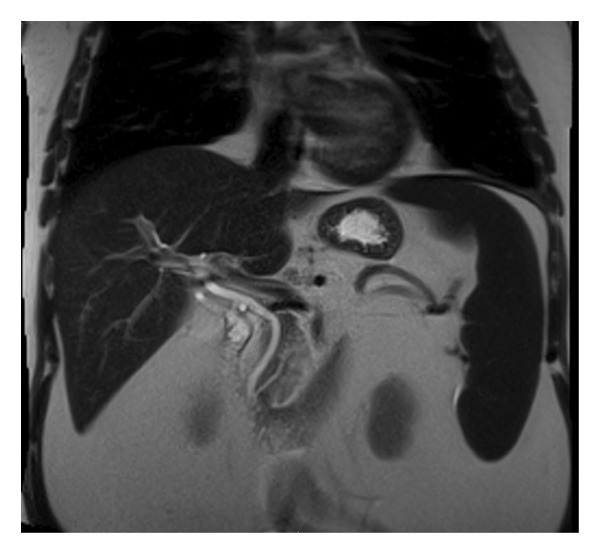
Coronal image from magnetic resonance cholangiopancreatography (MRCP) which reported no evidence of intra‐ or extrahepatic duct dilatation and normal‐calibre common bile duct and pancreatic duct.

Given the associated elevation in liver enzymes, the aetiology was felt to be hepatic or posthepatic. Considering the history of gallstones and hereditary spherocytosis, as well as presentation with right upper quadrant discomfort, obstructive jaundice secondary to gallstone disease was the leading differential diagnosis. The patient proceeded to have an urgent magnetic resonance cholangiopancreatography (MRCP). This reported several tiny calculi within the gallbladder and mild gallbladder wall thickening. However, the biliary tract was nondilated with no evidence of intraductal calculi or an obstructive lesion (Figure 1).

Substance use, drug induced injury and alcohol were considered. The patient had no history of alcohol excess, and the biochemical pattern, with AST not showing a 2:1 ratio over ALT, did not indicate occult alcohol consumption.

Vascular causes such as hepatic vein outflow obstruction were considered, although there was no clinical evidence of ascites to support this diagnosis. Triple‐phase CT of the liver showed splenomegaly (18 cm) without hepatic parenchymal abnormality, thrombosis or congestion, making a vascular cause unlikely.

A viral screen, including cytomegalovirus (CMV), adenovirus, Ebstein–Barr virus (EBV) and hepatitis A, B, C, E, was negative. The patient had no evidence of cutaneous varicella‐zoster virus and this was not tested for. Herpes simplex virus (HSV) was not tested for, given it is typically associated with hepatitis presentations in pregnancy, neonates or immunocompromised individuals. The patient had previously tested negative for human immunodeficiency virus (HIV). There was no rise in inflammatory markers or a history of fever to strongly suggest alternative infective aetiology.

The patient was investigated for immune‐mediated liver disease, in particular autoimmune hepatitis (AIH), although the cholestatic pattern (R value 1.2) was not typical of AIH. Immunology was unremarkable, including negative ANA, antimitochondrial antibody (AMA), antismooth muscle antibody (ASMA), anti–liver‐kidney microsomal antibody and low immunoglobulin *G* (IgG) 13.6 g/L. There was no suggestion of cholangiopathy such as primary sclerosing cholangitis on MRCP.

Genetic causes were sought. A 24‐h urinary copper collection was elevated at 1.65 (reference range < 10.60 μmol/24 h). However, there was no evidence of Kayser–Fleischer rings on slit‐lamp examination by ophthalmology, and there were no sequence changes of known significance in the ATP7B gene to suggest Wilson’s disease. Subsequent liver pathology demonstrated no evidence of copper overload, and so the Leipzig score was 0, suggesting an unlikely diagnosis of Wilson’s disease.

Genetic testing showed no evidence of a genotype associated with Gilbert syndrome.

An extended genetic panel was performed following discussion with clinical genetics to assess 32 genes associated with cholestasis (Table 1). No sequence changes of known significance were detected, and therefore an underlying genetic cause for his cholestasis presentation was not identified. In the absence of a family history of significant jaundice, whole genome sequencing was not indicated.

**TABLE 1:** Extended panel of genes associated with cholestasis.

Progressive familial intrahepatic cholestasis (PFIC)–associated genes
ATP8B1, ABCB11, ABCB4, TJP2, NR1H4, MYO5B, VPS33B
Alagille syndrome
JAG1, NOTCH2
Alpha‐1 antitrypsin deficiency mutation
SERPINA1
Dubin–Johnson syndrome
ABCC2
Bile acid synthesis and metabolism
AKR1D1, CYP7A1, CYP27A1, HSD3B7, BAAT, AMACR
Other (including disorders relating to peroxisomes, mitochondria, cholesterol and lipid trafficking, bilirubin metabolism)
ALDOB, BCS1L, CLDN1, DCDC2, FAH, NPC1, NPC2, PEX1, PEX12, PEX26, PEX6, SLC25A13, TALDO1, UGT1A1, VIPAS39

**FIGURE 2:**
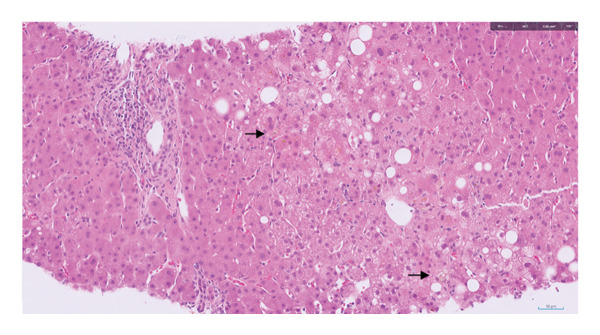
H&E‐stained histopathology section of the liver biopsy. There is a portal tract at the top left. The remainder of the tissue is parenchyma, which shows severe acute cholestasis (brown pigment, examples indicated by the arrows) and focal steatohepatitis.

A liver biopsy taken during acute admission showed severe acute cholestasis, mainly canalicular and perivenular (Figure 2). In addition, there was focal steatohepatitis; mild portal inflammation and bile ductular reaction; minimal focal deposition of copper‐associated protein in keeping with minimal chronic cholestasis histologically; and mild hepatocyte‐predominant iron deposition. Liver histology was discussed at the hepatology clinicopathology meeting. In view of the absence of interface hepatitis, absence of a predominantly lymphoplasmacytic infiltrate or rosetting of liver disease, the Revised International Autoimmune Hepatitis Group score was low at 3, suggesting ‘possible AIH’ only. There was no copper overload on formal quantification. The lack of prominent steatohepatitis reinforced the clinical impression that there was no role of alcohol in the patient’s presentation.

An endoscopic ultrasound of the biliary tract was performed as an urgent outpatient procedure. This demonstrated cholelithiasis but no evidence of ductal calculi or ductal abnormality.

## 4. Case Presentation: Treatment, Outcome and Follow‐Up

This case was managed conservatively with supportive measures including intravenous fluids to maintain hydration, antiemetics and simple analgesia. At Day 7 of inpatient admission, bilirubin and other liver biochemistry markers spontaneously began improving, and symptoms of nausea and abdominal discomfort resolved. After extensive investigation, a unifying diagnosis for the degree of hyperbilirubinaemia remained elusive. At Day 12, the patient was discharged from hospital with a plan to await liver biopsy results, genetic testing and urgent outpatient endoscopic ultrasound.

This patient remains under the care of hepatology and haematology, with regular follow‐up. At clinic review 5 months after admission, his liver biochemistry had returned to his previous longstanding baseline level of unconjugated hyperbilirubinaemia (70  μmol/L) with normal liver enzymes.

## 5. Discussion

### 5.1. A Case of Cholestasis of Unknown Origin

This case represented a diagnostic challenge for investigating jaundice in a patient with preexisting hyperbilirubinaemia. Other descriptions in the literature of profound jaundice in patients with a background of hereditary spherocytosis have been associated with concurrent Gilbert’s syndrome [4] or related to ductal stones [5]. We identified a case report describing two rarer cases of hereditary spherocytosis complicated by cholestasis; however, in both instances the patient ultimately developed overt sequelae of ductal stone disease in the form of pancreatitis [6]. There has been a case report of severe jaundice in hereditary spherocytosis associated with a gene variant predisposing to intrahepatic cholestasis (ABCB11) [7].

In the case we describe, Gilbert’s syndrome was excluded. The patient was also thoroughly assessed for ductal stone disease with two imaging modalities, both MRCP and endoscopic ultrasound. Neither modality demonstrated evidence of ductal stones. The degree of hyperbilirubinaemia was not typical of hereditary spherocytosis, and the patient was not anaemic. Genetic testing for variants associated with familial intrahepatic cholestasis was negative.

It is unclear if hereditary spherocytosis can be associated with idiopathic episodes of severe intrahepatic cholestasis, as seen in this patient.

An alternative explanation may be this that case represents a well‐established complication of hereditary spherocytosis which is the manifestation of biliary calculi and their potential for consequent biliary obstructive sequelae, although this was not demonstrated.

### 5.2. The Role of Splenectomy And Cholecystectomy in Hereditary Spherocytosis

The patient had previously declined surgery despite being offered splenectomy by his haematology team.

BSH guidelines recommend splenectomy based on disease severity, using a classification adapted from Eber et al. [8]. Splenectomy is recommended in severe hereditary spherocytosis, considered in moderate disease, and generally avoided in mild cases. Splenectomy reduces haemolysis and therefore symptoms and long‐term complications. Gallstones are cited as a ‘prime reason’ for splenectomy. However, this benefit must be balanced against increased risks of infection from encapsulated organisms and vascular events [9].

These recommendations are based on Grade C evidence. This highlights the need for more robust data, particularly regarding the role of splenectomy in patients with milder disease and asymptomatic gallstones, where the benefit remains unclear.

The role of cholecystectomy in the management of hereditary spherocytosis is complex and requires further evidence. As described, the condition is heterogeneous, and there will not be a one‐size‐fits‐all recommendation. Splenectomy should be sufficient if no gallstones are detected; the rationale being that this would eliminate the potential for future pigmented stone formation [10]. What is not known is how best to manage patients with proven gallstones, both asymptomatic and symptomatic.

Despite the initial high index of suspicion of gallstone‐related disease as the aetiology for the patient’s presentation, subsequent investigations did not suggest stones to be the cause. The cause of this profound hyperbilirubinaemia remains uncertain. It is possible that hereditary spherocytosis is associated with a phenomenon of idiopathic acute severe cholestasis. In the absence of any objective evidence of biliary obstruction and with self‐resolution of the cholestatic injury, the patient was advised that there was a lack of evidence to suggest that undertaking splenectomy and cholecystectomy in the past would have averted this presentation. In the absence of any confirmatory investigations for the cause of cholestasis, a watch‐and‐wait approach was taken.

### 5.3. Learning Points/Take‐Home Message

• The assessment of hyperbilirubinaemia in the context of preexisting unconjugated hyperbilirubinaemia can be challenging. Careful consideration of dual pathology is required, e.g., unconjugated hyperbilirubinaemia with further contribution of a competing biliary obstructive or intrahepatic cholestatic diagnosis.
• The aetiology of such profound hyperbilirubinaemia in this described case remains uncertain, but it raises the possibility that hereditary spherocytosis may be associated with idiopathic acute severe cholestasis. We report that this case was conservatively managed and self‐resolved.
• There is a recommendation to consider splenectomy and/or cholecystectomy in patients with hereditary spherocytosis and manifestations of biliary stone disease. However, despite the biological plausibility of these interventions, the evidence base to demonstrate benefit in terms of clinical outcomes on extended follow‐up appears to be less clear.

## Funding

No funding was received for this manuscript.

## Consent

No written consent has been obtained from the patient as there are no patient identifiable data included in this case report.

## Conflicts of Interest

The authors declare no conflicts of interest.

## Data Availability

Data sharing is not applicable to this article as no datasets were generated or analysed during the current study.
